# Nutritional Interventions to Improve Cachexia Outcomes in Cancer—A Systematic Review

**DOI:** 10.3390/medicina58070966

**Published:** 2022-07-21

**Authors:** Adina Braha, Alin Albai, Bogdan Timar, Șerban Negru, Săftescu Sorin, Deiana Roman, Dorel Popovici

**Affiliations:** 1 Second Department of Internal Medicine, “Victor Babeș” University of Medicine and Pharmacy, 300041 Timisoara, Romania; braha.adina@umft.ro (A.B.); bogdan.timar@umft.ro (B.T.); roman.deiana@umft.ro (D.R.); 2 Department of Oncology, “Victor Babeș” University of Medicine and Pharmacy, 300041 Timisoara, Romania; snegru@yahoo.com (Ș.N.); sorin.saftescu@umft.ro (S.S.); dorel.popovici@umft.ro (D.P.)

**Keywords:** cachexia, nutritional interventions, weight gain, eicosapentaenoic acid, β-hydroxy-beta-methyl butyrate, glutamine, appetite, cannabinoids

## Abstract

*Background and Objectives*: The prevalence of cachexia has increased across all of the cancer types and accounts for up to 20% of cancer-related deaths. This paper is a systematic review of nutritional interventions aiming to improve cachexia outcomes in cancer, focusing on weight gain. *Materials and Methods*: A search in Medline and Elsevier databases for articles up until the 23 January 2022, was conducted. *Results*: Out of 5732 screened records, 26 publications were included in the final analysis. Four randomized clinical trials showed a significant body weight (BW) increase in patients treated with eicosapentaenoic acid (EPA), β-hydroxy-beta-methyl butyrate (β-HMB), arginine, and glutamine or marine phospholipids (MPL). An upward BW trend was observed in patients treated with L-carnitine, an Ethanwell/Ethanzyme (EE) regimen enriched with ω-3 fatty acids, micronutrients, probiotics, fish oil, a leucine-rich supplement, or total parental nutrition (TPN) with a high dose of a branched-chain amino acid (BCAA). *Conclusions*: Although clinical trials relating to large numbers of nutritional supplements present promising data, many trials provided negative results. Further studies investigating the underlying mechanisms of action of these nutritional supplements in cancer cachexia are needed. Early screening for cancer cachexia risk and nutritional intervention in cancer patients before aggravating weight loss may stabilize their weight, preventing cachexia syndrome. According to the GRADE methodology, no positive recommendation for these nutritional supplements may be expressed.

## 1. Introduction

Cancer is one of the many chronic diseases associated with cachexia [1], a condition that leads to progressive dysfunction, high morbidity and mortality risk [2], and increased complications from cancer surgery [3]. In addition, it reduces the effectiveness of anti-cancer chemotherapy and increases chemotherapy toxicity [4], negatively affecting cancer patients’ quality of life (QoL) [5]. In 2014, 50–80% of cancer patients presented with cachexia, responsible for up to 20% of cancer deaths and 80% of mortality rates [4].

Skeletal muscle loss is the critical feature of cancer cachexia. The excessive catabolism from cancer triggers unintentional weight loss via skeletal muscle and adipose tissue loss, loss of appetite, reduced food intake, and high energy expenditure [5]. However, conventional nutritional support can partially reverse weight loss, but unfortunately, is it usually not entirely reversed [5].

Although first described in 1858 by ophthalmologist John Zachariah Laurence as a chronic wasting associated with malignant tumors [6], “cancer-related cachexia” received a formal definition only in 2011, in a publication by Kenneth Fearon [7]. In 2017, the European Society of Clinical Nutrition and Metabolism (ESPEN) defined cachexia as “chronic disease-associated malnutrition with inflammation”, showing that the cachexia pathology differs from starvation and malabsorption that does not include inflammation [8]. Cancer cachexia is different from starvation because of the impaired balance between skeletal muscle synthesis and breakdown and the increased resting energy expenditure (REE). Cachexia means the patient is eating and losing weight, while starvation means the patient cannot eat and thus loses weight [1].

Sarcopenia is another disease characterized by decreased skeletal muscle mass, strength, and function. However, its diagnosis requires evidence of low muscle mass, associated with either low muscle strength or low physical performance. Therefore, cachexia and sarcopenia must not be confused in patients with cancer. The difference lies in the underlying pathological processes leading to muscle wasting, represented by cancer-related inflammation in cachexia and age-related inflammation in sarcopenia [9]. 

The multifactorial pathogenesis of cachexia makes the diagnosis of this syndrome complicated and, consequently, difficult to define. Therefore, the evolving concept of cachexia results from a prolonged effort toward a definition of cachexia. In addition, Berardi et al. underlined that the term “cachexia” is often misused in fasting conditions, muscle disuse, and sarcopenia [10].

### 1.1. Pathophysiology

Cancer cachexia pathophysiology is multifactorial and includes chronic systemic inflammation, abnormal energy, and substrate metabolism changes. However, it is generally stated that the initial mechanism is represented by the production of pro-inflammatory cytokines by the tumor cells, such as interleukins (ILs), interferon-g, TNFa, and NF-kB. In addition, cancer causes the alteration of the protein, lipid, and glucose metabolism, which determine energy loss from food intake and the ineffective use of energy and substrates. Furthermore, the tumor cells “steal” nutrients from tissues for active replication [11].

The most significant changes in carbohydrate metabolism are represented by glucose intolerance, insulin resistance, accelerated glycogenesis, increased gluconeogenesis from lactate, and increased energy expenditure. There is an increased mobilization and oxidation of lipids for the lipid metabolism, resulting in consuming fat deposits. A tumor catabolic factor, lipid mobilizing factor (LMF), increases the energy expenditure and body fat loss by directly affecting the adipose tissue. TNF-a induces lipid depletion; IL-1, TNF-g, and IFN-g inhibit the lipoprotein lipase, stimulating lipolysis. Protein turnover is increased in protein metabolism in cancer cachexia, due to decreased hepatic and muscle tissue synthesis. The studies show a reduction in the gluconeogenetic amino acids in cancer patients’ plasma. Furthermore, the cancer cells secrete a proteolysis-inducing factor (PIF), which inhibits protein synthesis in the skeletal muscle, induces apoptosis, and increases proteolysis [12]. 

The chronic inflammation from the underlying disease, associated with aging and immobility, causes the anabolic resistance seen in the patients with cachexia. Furthermore, disease progression aggravates the mentioned metabolic abnormal changes and inflammatory response, worsening the anabolic resistance [13].

Recently, studies have shown the important role of the neuroendocrine system in cancer cachexia, especially that of the control centers of appetite: the hypothalamus, pituitary gland, and adrenal gland. On the one hand, orexigenic neurotransmitters promote appetite, such as the neuropeptide Y (NPY) and the agouti gene-related protein (AgRP). On the other hand, anorexigenic signals, such as proopiomelanocortin (POMC) and cocaine- and amphetamine-regulated transcript (CART) suppress appetite. In addition, the expression of the pro-inflammatory cytokines due to chronic inflammation affects the hypothalamus, causing the inactivation of NPY and AgRP and the activation of POMC and CART, resulting in anorexia, one of the main signs of cancer. Anorexia is also aggravated by symptoms such as pain, fever, dyspnea, diarrhea, depression, and delirium [14]. 

Based on recent data from the literature, McGovern et al. suggest that cancer-cachexia should be seen in perspective as an inflammatory systemic response syndrome that opens new horizons for cancer-cachexia treatment [15].

### 1.2. Assessment

A cancer cachexia assessment should include caloric intake, nutrition risk factors and symptoms, weight and body composition (BWC), weight change during the preceding months, performance status, biological markers, and information about body systemic inflammation [16]. According to Berardi and colleagues, the biomarkers used in clinical practice for assessing cachexia could be grouped into four categories: inflammation cytokines; lean muscle mass; markers of biological activity and altered metabolism; and other tumor factors [10]. In addition, several anorexia-cachexia signaling mediators, including activin A, myostatin, GDF15, and lipocalin-2, could be suitable for future targeted intervention [17].

Caloric intake is better evaluated through a dietary history collected prospectively, recording type, frequency, and quantity of meals. In addition, the symptoms that may affect caloric and nutrient intake, such as pain, nausea, vomiting, constipation, early satiety, alterations of taste or smell, and dysphagia, should be noted [18].

Evaluating the nutrition risk factors includes assessing cancer patients at risk for malnutrition. According to the ESPEN guidelines on nutrition in cancer patients, early nutritional screening is critical once a cancer diagnosis is established and should be repeated throughout treatment [19]. A malnutrition tool should identify the patients at risk, benefitting from nutritional intervention and initiate a specific action and consecutive nutritional care plan. Unfortunately, there is no “gold standard” among malnutrition tools. Until now, thirty-two screening tools have been developed, of which twenty-four assess the patients’ nutritional status, four aim to predict clinical outcomes, and four do both [19]. The most commonly used screening tools are:

- Patient-Generated Subjective Global Assessment (PG-SGA) [20];
- Mini Nutritional Assessment (MNA) [21];
- Malnutrition Universal Screening Tool (MUST) [22];
- Nutritional Risk Screen (NRS-2002) [23];
- NUTRISCORE [24];
- Weight Loss Grading System (WLGS 0-4) [25].

The Oncology Nutrition Dietetic Practice Group of the American Dietetic Association adopted the PG-SGA as the standard nutritional screening tool for patients with cancer, which are also recommended in the UK guidelines.

The biological markers linked with cachexia include elevated C-reactive protein (CRP) and low serum albumin. Based on these laboratory abnormalities associated with malnutrition, some prognostic scoring tools have been developed: Prognostic Inflammation Nutrition Index (PINI); Nutritional Risk Index (NRI); and the modified Glasgow Prognostic Score, which correlates with a decreased response to chemotherapy and a higher sensitivity to toxicities [26]. Other laboratory findings may include high neutrophil/lymphocyte ratio, hyperglycemia, hypertriglyceridemia, and insulin resistance

The assessment of energy balance includes an evaluation of the total energy expenditure (TEE). TEE is represented by two components: energy consumed by physical activity; and REE. REE can be measured by indirect calorimetry, or it can be predicted with the Harris–Benedict equations, which account for sex, height, weight, and age. TEE is rarely measured since it requires specialized equipment and expertise. However, accurate REE measurements allow healthcare workers to be prevented from under- or overfeeding cancer patients [27]. Because of REE, cancer patients have increased energy costs due to disease burden, altered BWC, and chronic inflammation.

The BWC may be assessed through anthropometry (body weight (BW), BMI, body surface, skinfold measures), computer tomography (CT), and dual-energy X-ray absorptiometry (DXA). The anthropometry measurements are less accurate than CT, and DXA, due to the inability to distinguish lean muscle mass and fat tissue. DXA and CT are the gold standards for BWC assessment, with precision and specificity for the tissue components [16]. 

### 1.3. Diagnosis Criteria and Staging

There are a series of debates regarding the diagnostic criteria of cancer cachexia. With the proper diagnostic criteria, better identification of the cachexia patient in the early stages may guide clinical and therapeutic decisions.

In 2011, Fearon et al. defined cachexia as weight loss of 5% or more within 6 months; weight loss of 2% or more in patients with a body mass index (BMI) < 20 kg/m^2^; or weight loss of 2% or more in patients with sarcopenia [7]. This definition proposed by the international consensus is used today. In addition, the same group formulated three stages of cancer cachexia: pre-cachexia; cachexia; and refractory cachexia, based on assessing food intake, catabolic disturbances, functional and psychosocial impact, and BWC. In addition, it was recommended to use BMI and degree of weight loss to grade the severity of the weight loss [7]. 

In 2016, the Global Leadership Initiative on Malnutrition (GLIM) proposed diagnostic criteria for malnutrition, also recommended by the European Society for Medical Oncology (ESMO) and used in their practice guidelines for cancer cachexia in adult patients. The proposed framework consists of three phenotypic criteria (involuntary weight loss, low BMI, reduced muscle mass) and two etiologic criteria (reduced food intake or assimilation, and disease burden or inflammatory condition). The diagnosis of malnutrition requires at least one phenotypic and one etiologic criterion to be met [28]. 

In addition, in 2011, Argiles et al. developed “the cachexia score” (CASCO). This staging system divides cancer patients according to the cachexia severity, which helps guide the type of treatment. In addition, the staging system assesses body weight loss and BWC, inflammation/metabolic disturbances/immunosuppression (IMD), physical performance (PHP), anorexia (ANO), and QoL, and it classifies cachexia into mild, moderate, severe, and terminal [29].

In 2018, a cachexia staging score (CSS) for patients with advanced cancer was developed and validated to clarify the three staging systems, assessing: weight loss in 6 months (0–3 points); SARC-F (a questionnaire used as a screening tool for sarcopenia, 0–3 points); ECOG performance status (0–2 points); appetite loss (0–2 points); and abnormal biochemistry (0–2 points). In addition, the CSS divides the patients into four groups: non-cachexia (0–2 points); pre-cachexia (3–4 points), cachexia (5–8 points), and refractory cachexia (9–12 points) [30]. 

In 2017, ESPEN published evidence-based guidelines for nutritional care and recommended:

- To screen all of the patients with cancer for nutritional risk as soon as possible, regardless of body mass index and weight history;
- To include multiple nutrition-related assessment practices: measures of anorexia, BWC, inflammatory biomarkers, resting energy expenditure (REE), and physical function;
- To implement multimodal nutritional interventions with individualized plans [31]

In a recent review of the nutritional management of cancer cachexia, Tanaka and his team analyzed the effect on cancer cachexia of many nutrients contained in blue fish and red meat. They suggested consuming these nutrients would counteract muscle loss due to cancer cachexia [32]. The current systematic literature review aims to identify relevant evidence, using bibliographic databases for a critical evaluation of the efficacy and pathogenesis of nutritional interventions in improving cachexia outcomes in patients with cancer.

## 2. Materials and Methods

For the review purpose, the primary outcome was any weight change or improvement in nutritional and performance status in cancer cachexia patients. 

### 2.1. Eligibility Criteria

The review included full-text papers or abstracts of clinical trials on human subjects comparing nutritional interventions in cancer cachexia. The publications reporting on animal subjects, non-cancer cachexia, or case reports were not eligible for this review. The nutritional interventions reviewed referred to vitamins, minerals, fatty acids, proteins, or other dietary supplements. 

### 2.2. Information Sources

The eligible publications were recorded through electronic searching in Medline (PubMed) and Elsevier (ClinicalKey) databases, respectively, by manual searches through reference lists of the recorded articles from inception until 23 January 2022. 

### 2.3. Search Strategy

The search strategy was restricted to English publications and involved combining two strings: cancer cachexia and nutrition, OR N-3 polyunsaturated fatty acids OR omega-3, OR supplement, OR vitamin, OR minerals, OR carotenoids, OR flavonoids, OR macronutrient, OR fibers/prebiotics. 

### 2.4. Selection Process

The list of identified articles was exported to a citation manager, EndNote, which automatically removed the duplicates to avoid reviewing duplicate articles. We retrieved in full all of the studies with an abstract referring to the subject of nutrition, supplement, vitamins, vitamin D, minerals, lipids, carbohydrates, protein, fatty acids, N-3 polyunsaturated fatty acids/omega-3, fish oil, carotenoids, flavonoids, fibers/prebiotics aimed at improving cancer cachexia outcome in human subjects. 

### 2.5. Data Collection Process and Data Items

**Figure 1:**
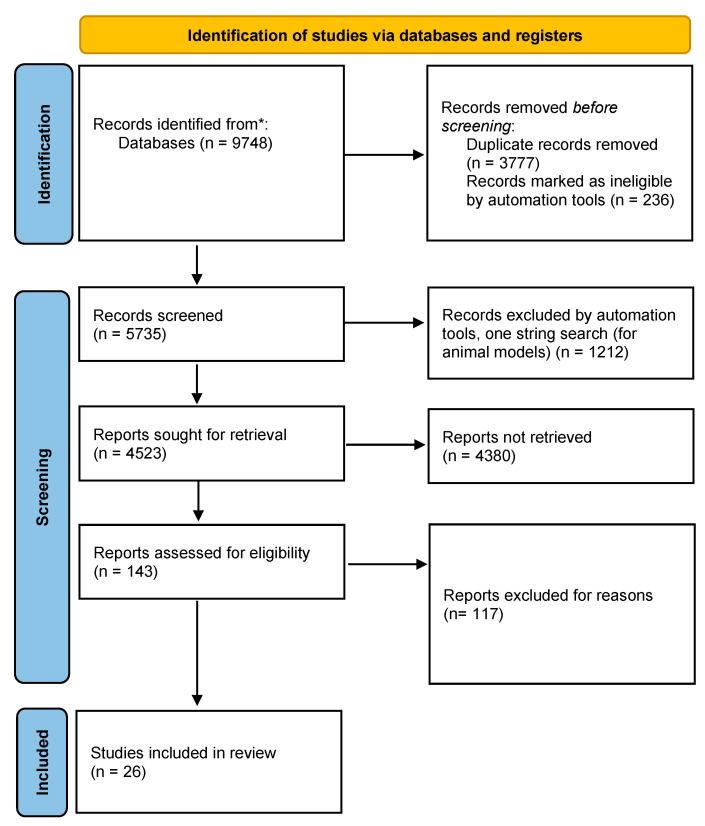
Study flow diagram. * Medline (PubMed) and Elsevier (ClinicalKey) databases, manual searches through reference lists of the recorded articles.

Two of the authors extracted the data (Figure 1), using the PRISMA flow diagram [33] and reviewed the data from the records. Three of the authors cross-checked the findings in a second step. Finally, four of the authors cross-checked a sub-sample. We resolved disagreement by consensus.

### 2.6. Study Risk of Bias Assessment

Two of the authors independently assessed the risk of bias using the Cochrane RevMan 5.3 software (The Cochrane Collaboration, London, United Kingdom) [34], following the Cochrane Handbook for Systematic Reviews of Interventions [35]. Any disagreements were discussed and analyzed with a third author. 

Each study was screened for possible selection, detection, or attrition bias. The studies had a low risk of selection bias if the method used to generate the allocation sequence to conceal allocation to the intervention was clearly described. Otherwise, the risk of selection was unclear. In addition, if the investigators or participants were blinded, the risk for performance was low. In addition, if the outcome assessment was stated as blinded, the risk of detection bias was low, and the risk was unclear if the outcome assessment was not described. We did not identify incomplete outcome data or reporting bias. 

The included studies were checked for possible biases confounded by other biases, such as the small size of the treatment arm: low risk of bias (at least 200 participants); unclear risk of bias (50–199 participants); and high risk of bias (fewer than 50 participants).

**Figure 2:**
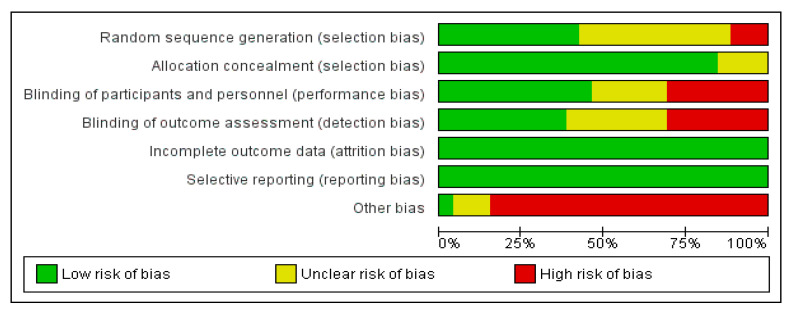
Risk of bias graph: review authors’ judgments about each risk of bias item presented as percentages across all included studies.

**Figure 3:**
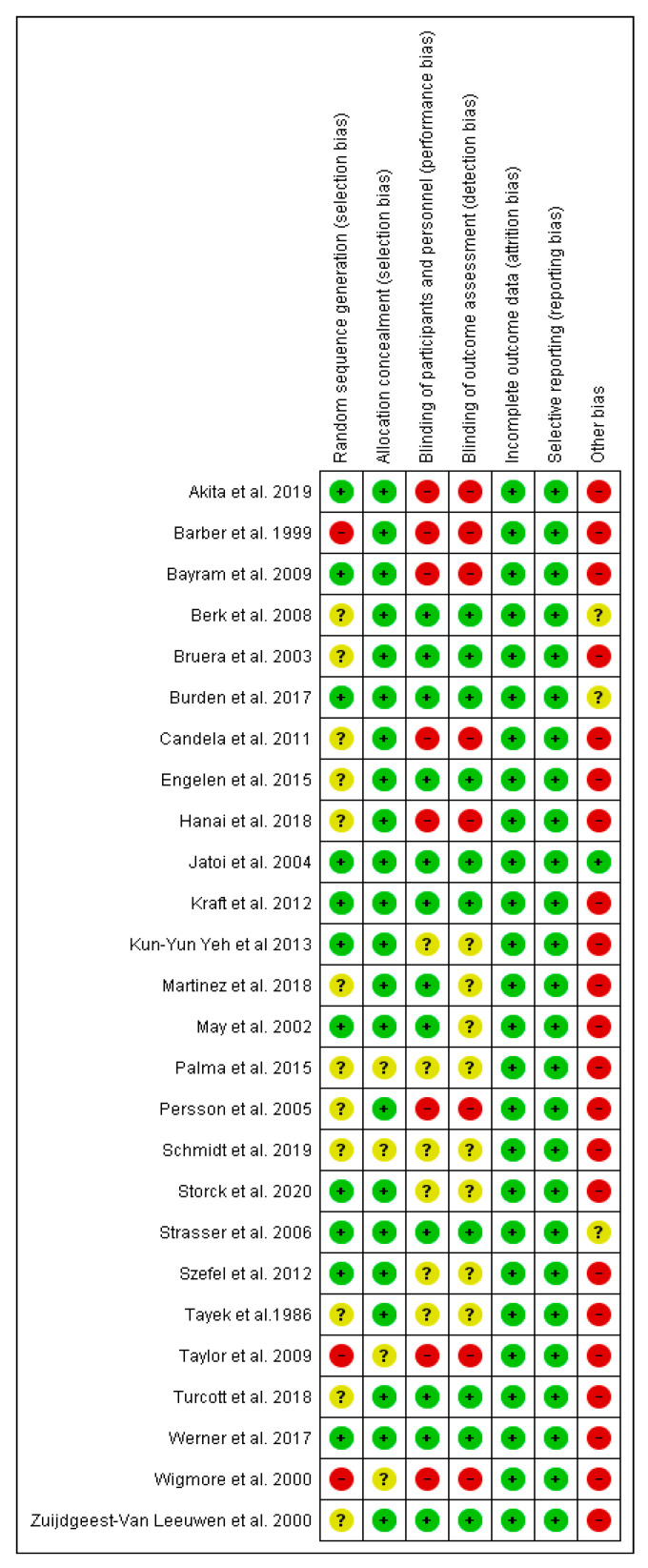
Risk of bias summary: review authors’ judgments about each risk of bias item for each included study.

The risk of bias graph and summary are presented in Figure 2 and Figure 3. In addition, the review authors’ judgments about each risk of bias item for each of the included studies are presented in the Supplementary Materials.

## 3. Results

### 3.1. Study Selection

We screened 5735 publications, out of which 143 reports were assessed for eligibility after reading the title and abstract. Finally, 26 studies [36,37,38,39,40,41,42,43,44,45,46,47,48,49,50,51,52,53,54,55,56,57,58,59,60,61] that met the inclusion criteria and the quality assessment request were included in the analysis.

### 3.2. Study Characteristics

The clinical trials included were designed in a non-randomized or randomized manner. One report referred to a pediatric population of patients affected by different types of cancer. The rest of the studies referred to an adult population affected by cancer: pancreatic, colorectal, non-small cell lung, head and neck squamous cell, solid tumors, gastrointestinal (GIC), and metastatic intra-abdominal cancer. The study durations ranged from 0 days to 24 weeks. In addition, the interventional arms included a small number of patients ranging from 9 to 235.

The outcome measures across the trials were: improvement of skeletal muscle mass (SMM) and psoas major muscle area (PMA) before and after chemoradiotherapy (CRT); weight changes; body composition; nutritional and performance status; QoL; appetite; fatigue; biological parameters; inflammation markers; protein anabolism; compliance to the nutritional intervention; complications.

The nutritional intervention implied the administration of oral supplements (tablets or liquid) or total parenteral nutrition (TPN) containing: L-carnitine; leucine; branched-chain amino acid (BCAA); cannabinoid extract (CE); essential amino acids (EAA); Ethanwell/Ethanzyme (EE); eicosapentaenoic acid (EPA); fish oil (FO); ω-3 fatty acids; Guarana; megestrol acetate (MA); medium-chain triglycerides (MCTs); marine phospholipids (MPL); oleic acid (OA); cannabinoids; oral nutrition supplementation (ONS); delta-9-tetrahydrocannabinol (THC); total parenteral nutrition (TPN); β-hydroxy-beta-methyl butyrate (βHMB); a combination of HMB, arginine, and glutamine. The reported adverse effects of the interventions were not significant. In the Akita et al. study [36], the authors reported that 17 of the patients consumed less than 50% of the provided EPA supplement, and eight patients did not drink it at all because of the taste. In another study by Burden et al. [41], the patients complained about burping and a bad aftertaste after drinking the protein supplement. In the Berk et al. study [39], patients complained about nausea, constipation, or diarrhea; others withdrew from the study without explained reasons. The CE and THC supplements were associated with severe adverse effects, mainly dizziness, nausea/vomiting, and dyspnea [54].

The study characteristics are presented in Table 1. The dynamics of the patients’ body weight as a dichotomic event after nutritional intervention in the studied clinical trials are presented in Table 2.

### 3.3. Risk of Bias in Studies

Less than 50% of the analyzed studies had a low risk of randomization, and most had a low allocation risk. About half of the studies showed a low risk of performance and detection bias. The risk of attrition and reporting bias was low among all of the studies. However, most of the studies included less than 50 participants in the experimental arm, determining a high risk of bias deriving from the small size of the treatment arm (as shown in Figure 2 and Figure 3).

### 3.4. Results of Individual Studies

Six trials [36,44,54,55,58] involving supplementation with EPA, ω-3 fatty acids, CE and THC, TPN, LCTs, LCTs plus MCTs, or Nabilone reported a decrease in BW after the nutritional intervention. However, only one study [36] showed a significant decrease in BW after five weeks of EPA supplementation in 31 cachectic patients with pancreatic cancer. However, the EPA supplementation in these patients was associated with an increased post/pre ratio of SMM, depending on the supplement dose (*p* = 0.02). The post/pre ratio of PMA in the NI group was significantly higher than that of the normal diet (ND) group. In the Bayram et al. study, 33 pediatric patients aged 7.7 ± 2.7 years that received EPA failed to gain weight. However, the study showed a decrease in the percentage of patients with weight loss in BW (6.1% vs. 47.4%; *p*= 0.001), BMI (12.1% vs. 52.6%; *p*= 0.002), and a negative deviation in weight percentile (6.1% vs.31.6%; *p*= 0.021) [38]. In two other studies, the patients receiving EPA [45] or ω-3 fatty acids [44] showed a downtrend in BW without statistical significance.

Eight trials failed to show a significant change in BW measurements. Three of them involved EPA administration [42,45,48], as shown in Table 2. The studies, which included supplements with FO [40,52,59], ONS [41], or Guarana [50], also did not lead to a significant change in patient weight.

However, four RCTs showed a significant increase in BW in the patients treated with EPA [37,60], β-HMB, arginine, and glutamine [49] or MPL [57]. While the patients who were treated with L-carnitine [46], an EE regimen enriched with ω-3 fatty acids, micronutrients, probiotics [47], FO [48], a leucine-rich supplement [53], or TPN with a high dose of BCAA [56], showed an upward trend of BW.

Another study showed that high leucine levels in the EAA/Leucine mixture were of no anabolic benefit in patients with advanced non-small-cell lung cancer [43]. In another study comparing the effect of EPA-EE or OA-EE on BW, no differences were found in the weight loss percentages after intervention [61].

## 4. Discussion

Many clinical trials were found involving dietary supplements used to treat cancer cachexia. However, only 26 studies were analyzed in this systematic review. The main focus of this review was to evaluate the weight dynamics in patients with cancer cachexia treated with different dietary supplements.

Most of the studies were designed in a randomized fashion and included both a control arm and an experimental arm. However, the experimental arm in most of the studies only included a small number of patients. Some of the patients were lost at follow-up, and others were withdrawn from the studies. Therefore, we considered more than 75% of the included studies to be at a high risk of bias, due to the small number of treated patients with dietary supplements.

The effects on weight gain in the cancer cachectic patients treated with EPA are inconclusive. In a study [36], the patients undergoing neoadjuvant CRT for pancreatic cancer received two bottles/day of EPA-enriched nutrition drinks or a normal diet and showed a significant decrease in weight before and after the CRT in both of the groups. In addition, the post/pre ratio of skeletal muscle mass was similar in both of the groups. However, it is important to mention that only 45% of the patients in the experimental group managed to drink more than 50% of the EPA-enriched nutrition drinks, and they showed a significant increase in the PMA ratio.

In the Barber et al. study [62], the patients received two cans/day of a fish oil-enriched nutritional supplement, containing 1.09 g EPA. The study showed significant weight gain at both the 3- (median 1 kg, *p* = 0.024) and 7-weeks (median 2 kg, *p* = 0.033) follow-up. However, the effect on BW of this nutritional intervention may be overestimated and biased, due to the lost patients at the 3- and 7-week follow-ups (*n* = 18/20, respectively *n* = 13/20).

The percentage of the pediatric patients showing weight loss at the end of the 3-month follow-up in the Bayram et al. study was significantly lower in the patients treated with two containers of an oral supplement containing proteins and 1.09 g of EPA than in the control. However, when comparing the BMI of both groups at 6-month follow-up, there were no differences [38]. The neutral effect on weight, BMI, and body composition was also demonstrated by Candela et al., where patients received 1.5 g of EPA supplements for one month [42]. In a clinical trial by Jatoi et al., 1.09 g/bid of EPA supplements were neutral compared to MA on the outcome of gaining weight at least 10% from the baseline [45], but the effects of MA could have masked the EPA effects, due to the absence of a pure placebo arm. In another study by Martinez et al., patients received 2 g of EPA supplements or a placebo. The patients in the experimental arm showed a stabilization of weight compared to the placebo group, who showed a mean 2 kg of weight loss [48]. In the Hanai et al. study [44], the patients also received about 2 g of EPA in a supplementation drink daily but failed to show a gain in weight.

The Wigmore et al. study [60] contained high-purity EPA, with weekly increased doses from 1 g/day/first week, 2 g/day/second week, 4 g/day/third week, to 6 g/day from weeks 4–12. The results of this study were the most promising, showing a significant and stable weight gain of 0.5 kg after four weeks of EPA supplementation, without changes in the TBW. These results suggest that the beneficial effects of EPA-enriched nutrition drinks may be dose-dependent.

In addition, a small group of patients treated with FO or melatonin showed an upward trend of weight gain, but with a low clinical significance. The weight gain in this study might also be due to the dietary advice provided to the analyzed patients [51]. The patients from the Bruera et al. study were evaluated after a brief treatment period with FO and showed no weight gain or appetite improvement [40].

In another study, only 50% of the patients treated with FO showed an increase in weight compared to the control group; for some of the patients this was due to the increase in water content. Nevertheless, the BMI was similar in the two groups at the end of the 6-week study [59]. In a clinical trial, the patients who received three times a day capsules, containing 18.8 g/100 g EPA and 22.8 g/100 g DHA bound in neutral lipids and 16.5 g/100 g EPA and 33.7 g/100 g DHA bound in phospholipids, showed a significant weight gain at the end of the 6-week trial [57]. Compared to the previous study, where the MPL capsules contained 35% of ω-3-FA phospholipids (mainly phosphatidylcholine) plus 65% of neutral lipids (8.5 g/100 g EPA and 12.3 g/100 g DHA), 47% of the patients gained weight [59]. Oral nutritional supplementation with an EE regimen enriched with ω-3 fatty acids, micronutrients, and probiotics stabilized BW in patients with severe cachexia during the first four weeks of treatment [47]. However, severe diarrhea may limit the use of such a dietary formula.

The underlying mechanisms of the EPA in cancer cachexia are not fully understood. However, recent evidence from the literature showed that the EPA attenuates the protein degradation, lipid mobilization, and reduced glucose consumption in skeletal muscle induced by a proteolysis-inducing factor [63,64,65]. The EPA inhibits the protein catabolism by an ATP-dependent proteolytic pathway and the downregulation of proteasomes in the cachexia-inducing tumor caused by a proteolysis-inducing factor [66]. This proteasome pathway is upregulated by a transcription factor NF-κB, and EPA reduces the nuclear migration of NF-κB [67]. In addition, the studies in vitro showed that EPA reduces the levels of TNF-α and contributes to reduced muscle mass loss [68].

EAA/Leucine supplementation may also play a role in preventing muscle loss in cancer cachexia. This role is suggested by the linear relationship between net protein anabolism and the amount of EAA available in the systemic circulation found in the Engelen et al. study [43], but further studies are needed to explain this relationship.

The supplementation with β-HMB, arginine, and glutamine showed benefits in weight gain [39,49]. However, in one study [39], only 37% of the patients completed the study due to low compliance, adverse effects, or study withdrawal. In the other study [49], the patients receiving supplementation with the HMB/Arginine/Glutamine for four weeks showed a weight gain with a mean of 0.95 kg, based on a significant increase in fat-free mass (FFM).

The preliminary data from the CARPAN study [46] showed a beneficial effect of 4 g of L-Carnitine supplementation on weight gain after 12 weeks of treatment. However, future studies of a higher statistical power are needed to confirm this result. On the other hand, a group of patients treated with high-leucine supplements failed to show significant improvements in nutritional status, but improved handgrip strength [53].

Regarding CE and THC supplementation, no differences between the groups were observed in increase in appetite or BW change or weight loss (average, 600 g) at baseline or week 6 [54]. Moreover, CE and THC to improve cancer cachexia outcomes may be limited, due to the multiple adverse events. Nabilone, a synthetic analog of THC used in the Turcott et al. study, showed a significant weight loss in the experimental arm. However, the overall weight change was similar in both of the groups. The supplementation with Nabilone in cancer-cachexia may, however, safely increase the energy intake [58]. Only two patients showed a weight gain in a pilot study with Guarana supplementation over a 4-week trial by Palma et al. [50]. Others proved to be stable in weight, and increased appetite. The results of this study are contradictory to other pieces of evidence in the literature. Therefore, no recommendations for general use could be expressed.

Although the Szefel et al. study was not designed to investigate weight gain after TPN with LCTs, or LCTs plus MCTs, it proved that the skeletal muscle concentration in L-Carnitine is a true reflection of L-Carnitine deficiency in cancer-cachectic patients, and not plasma or urine levels. In addition, TPN with MCT/LCT lipid emulsion reduces the degradation of skeletal muscles in comparison with the pure LCT emulsion [55]. In a trial by Tayek et al., the patients treated with conventional TPN (19% BCAA) and a BCAA-enriched TPN formula (50% BCAA) showed an improvement in nutritional status by acting on protein metabolism [56]. The BCAAs supplementation may decrease proteolysis and increase protein synthesis in skeletal muscle by activating the mTOR pathway and inflammation modulation through glutamine production [69,70].

Although large numbers of clinical trials for nutritional supplements present promising data, many trials give negative results. In addition, the study designs are heterogeneous, have different study follow-ups, and have insufficient statistical power, which may influence the clinical outcomes. In addition, future studies are needed, investigating the underlying mechanisms of action of these nutritional supplements in cancer cachexia.

## 5. Conclusions

Early screening for cancer cachexia risk, and nutritional intervention in cancer patients before aggravating weight loss, may stabilize their weight, preventing cachexia syndrome. According to the GRADE methodology, no positive recommendation for the nutritional supplementation with essential amino acids, L-carnitine, branched-chain amino acid, ω-3 fatty acids, Guarana, cannabinoids, β-hydroxy-beta-methyl butyrate, a combination of HMB, arginine, glutamine and total parenteral nutrition could be expressed. However, no serious adverse effects were reported. Further research is needed to identify the efficacy relating to weight gain and the safety of these supplements in cachectic patients, to provide clear evidence-based recommendations.

## Figures and Tables

**Table 1:** The characteristics of the studies included in the analysis.

Study ID	Design	Type of Cancer	*n* *	Intervention	Duration	Outcomes	Results
Akita et al., 2019 [36]	RCT	Pancreatic	31	EPA	5 weeks	Improvement of SMM and PMA before and after CRT	The post/pre ratio of SMM in the nutritional intervention (NI) group increased with increases in supplement intake (*p* = 0.02). The post/pre ratio of PMA in the NI group was significantly higher than that of the normal diet (ND) group
Barber et al., 1999 [37]	CT	Pancreatic	20	EPA	7 weeks	Weight gain (BWC, dietary intake, REE and performance status)	Weight-gain at both 3 (median 1 kg, *p* = 0.02) and 7 weeks (median 2 kg, *p* = 0.03). Dietary intake increased significantly by almost 400 kcal per day (−1) (*p* = 0.002). REE per kg BW and lean body mass (LBM) fell significantly. Performance status and appetite were significantly improved at 3 weeks.
Bayram et al., 2009 [38]	RCT	Pediatric	33	EPA	24 weeks	BW, BMI, and weight percentile	Decrease in % of patients with weight loss in BW (6.1% vs. 47.4%; *p* = 0.001), BMI (12.1% vs. 52.6%; *p* = 0.002), and a negative deviation in weight percentile (6.1% vs.31.6%; *p* = 0.021)
Berk et al., 2008 [39]	RCT	Various	235	3 g of HMB, 14 g arginine, and 14 g of glutamine	8 weeks	LBM, body plethysmography, weight, the Schwartz Fatigue Scale, and the Spitzer QoL Scale	No statistically significant difference
Bruera et al., 2003 [40]	RCT	Various, advanced	46	FO	12 days	Appetite, tiredness, nausea, well-being, caloric intake, nutritional status, and function were prospectively assessed on days 1 and 14	No significant influence on appetite, tiredness, nausea, well-being, caloric intake, nutritional status, or function after two weeks
Burden et al., 2017 [41]	RCT	Colorectal	55	250 mL/day ONS and dietary advice	1 week	% weight loss, total complications, and BWC measurements	Less weight loss following surgery for colorectal cancer
Candela et al., 2011 [42]	RCT	Various	16	EPA	4 weeks	Anthropometric and biological parameters, QoL (SF-36 questionnaire)	No significant changes in anthropometric and biological parameters except significantly decreased interferon gamma (INF-γ) values (0.99 ± 0.95 vs. 0.65 ± 0.92 pg/mL, *p* < 0.05
Engelen et al., 2015 [43]	RCT	Advanced non-small-cell lung cancer	13	EAA, high leucine mixture	-	BW, height, fat, fat-free mass (FFM), respiratory muscle function, handgrip strength, and endurance. Protein anabolism	High leucine levels in the EAA/Leucine mixture were of no anabolic benefit. A highly significant linear relationship between net protein anabolism and the amount of EAA available in the systemic circulation (R (^2^): 0.85, *p* < 0.001) was found in both groups.
Hanai et al., 2018 [44]	RCT	Head and neck squamous cell carcinoma	14	ω-3 fatty acids	28 days perioperative	Weight, lean body mass, albumin, prealbumin, CRP, IL6, white blood cell count, body temperature, postoperative complications	Not effective for maintaining the nutritional status
Jatoi et al., 2004 [45]	CT	Various	421 in 3 arms	EPA versus MA, or both	4 weeks	A 10% weight gain above baseline	No improvement in weight or appetite
Kraft et al., 2012 [46]	RCT	Pancreatic	38	L-carnitine	12 weeks	Adverse effects, QoL, fatigue, BMI, BWC, survival time, L-carnitine level, CRP, CA 19-9, albumin, leucocytes	Body-mass-index increased by 3.4 ± 1.4%; nutritional status (body cell mass, body fat) and QoL parameters improved
Kun-Yun Yeh et al., 2013 [47]	RCT	Head and neck cancer	31	EE regimen enriched with ω-3 fatty acids, micronutrients, and probiotics	12 weeks	BW changes, serum albumin and prealbumin levels	Significantly increased BW and maintained higher serum albumin and prealbumin levels
Martinez et al., 2018 [48]	RCT	Head and neck squamous cell cancer	32	EPA	6 weeks	BWC, IL-1β, IL-6, TNF-α and IFN-γ, CRP, serum proteins, and blood count	A decrease in serum levels of IL-1β, IL-6, TNF-α, and IFN-γ, and regulation of BW (−0.3 ± 5.9 vs. −2.1 ± 3.7), LBM (−0.2 ± 3.8 versus −1.3 ± 3.6), BFM (0.2 ± 3.5 vs. −1.2 ± 3.8), and QoL (10 ± 33 vs. 5 ± 34).
May et al., 2002 [49]	RCT	Solid tumors	18	β-HMB, arginine, and glutamine	24 weeks	Change in body mass and fat-free mass (FFM)	Weight gain of 0.95 +/− 0.66 kg in 4 weeks, significant FFM increase of 1.12 +/− 0.68 kg
Palma et al., 2015 [50]	CT	Various, advanced	18	Guarana (Paullinia cupana)	4 weeks	A positive response in the first phase to be at least 5% weight gain or a three-point improvement in the appetite scale in at least three of the first 18 evaluable patients	Only two patients had weight gain above 5% from their baseline, whereas six patients had at least a three-point improvement in the visual appetite scale; a significant decrease in the lack of appetite and in somnolence
Persson et al., 2005 [51]	RCT	Advanced GIC	13	FO and melatonin	4 weeks	Tumor necrosis factor-alpha, IL-1β, soluble IL-2 receptor, IL-6, IL-8, and EPA, DHA, arachidonic acid, and linoleic acid.	No major changes in biochemical variables and cytokines were observed with any intervention. In the FO group, 5 of 13 patients (38%) showed weight stabilization or gain compared with 3 of 11 patients (27%) in the MLT group.
Schmidt et al., 2019 [52]	CT	GIC	13	FO	4 weeks	Acceptability and compliance, nutritional status and side effects, leukocyte, platelet counts, and markers of dose-limiting toxicities of chemotherapy.	FO in capsules appeared to result in better compliance than a nutritional drink with an equivalent dose of ω-3 LC PUFAs. There were no differences between the groups concerning changes in whole blood ω-3 LC PUFAs, weight, nutritional status, acceptability, or side effects; in the capsule group, the whole blood ω-3 LC PUFAs correlated negatively with the increase in nausea. No changes in median thrombocyte or leukocyte blood counts were observed.
Storck et al., 2020 [53]	RCT	Various, advanced	27	Leucine-rich supplement (whey protein)	12 weeks	Physical function, physical performance tests, nutritional status, dietary intake, fatigue, QoL, and clinical course	The secondary endpoint handgrip strength improved significantly. No significant differences between the other outcomes
Strasser et al., 2006 [54]	RCT	Various, advanced	164	CE and THC	6 weeks	Appetite, mood, and nausea, QoL CE-related toxicity was assessed every 2 weeks.	Increased appetite was reported by 73%, 58%, and 69% of patients receiving CE, THC, or PL, respectively.
Szefel et al., 2012 [55]	RCT	Various	25	TPN with LCTs, or LCTs plus MCTs as 50/50.	10 days	L-Carnitine distribution and the effects of parenteral lipid emulsions on plasma L-Carnitine levels and urinary excretion	A diet of MCTs/LCTs reduces L-carnitine release from muscle to plasma and urine more effectively than LCTs.
Tayek et al.,1986 [56]	RCT	Intra-abdominal metastatic adenocarcinoma	10	Conventional TPN (19% BCAA) and a BCAA-enriched TPN formula (50% BCAA)	-	Changes in the whole-body leucine kinetics and fractional rates of albumin synthesis	BCAA-enriched formulas improve whole body leucine kinetics, fractional rates of albumin synthesis, and leucine balance, and thus may favorably influence protein metabolism in cancer cachexia.
Taylor et al., 2009 [57]	CT	Various	17	MPL	6 weeks	Compliance, changes in BW, appetite, and QoL, fatty acid profile in plasma and blood cells	Significantly reducing the ω-6 to ω-3 fatty acid ratio, median weight change of +0.6% after 6 weeks), while appetite and QoL improved.
Turcott et al., 2018 [58]	RCT	Advanced non-small cell lung cancer	9	Drugs derived from cannabinoids (Nabilone)	6 weeks	Appetite, nutritional status, and QoL	Increased caloric intake (342 kcal), and significantly higher intake of carbohydrates (64 g) compared to patients receiving PL (*p* = 0.040). QoL also showed significant improvements
Werner et al., 2017 [59]	RCT	Pancreatic	31	FO, MPL	6 weeks	Routine blood parameters, lipid profiles, BW, and appetite	50% of the FO group gained BW during the 6-week intervention. In the MPL group, 47% gained weight; no significant change in fat mass, muscle mass, and body water; no significant statistical difference in BMI in both groups
Wigmore et al., 2000 [60]	CT	Advanced pancreatic cancer	26	High-purity EPA	12 weeks	Overall survival, changes in BWC, hematologic and clinical chemistry variables, acute-phase protein response, and performance status	A median weight gain of 0.5 kg persisted over the 12-week study period. TBW as a % of BW remained stable, as did the % of patients with an acute-phase protein response, nutritional intake, and performance status. Overall median survival from diagnosis was 203 days.
Zuijdgeest-Van Leeuwen et al., 2000 [61]	RCT	Various	17	EPA-EE (6 g/d) or PL OA-EE; 6 g/d)	7 days	Whole-body lipolysis and palmitic acid release were measured in the overnight fasting state, changes in weight, plasma free fatty acids (FFA), triacylglycerols (TAG), CRP, albumin, and prealbumin	No significant effects of EPA-EE on whole-body lipolysis, palmitic acid release, or palmitate oxidation were detected in cancer patients or healthy subjects compared to OA-EE. EPA-EE reduced plasma-FFA and TAG concentrations significantly in healthy subjects but not in cancer patients.

Abbreviations: *n* * = number of patients included in the experimental arm; % = percentage; BCAA = branched-chain amino acid; BFM = body fat mass; BW = body weight; BWC = body weight composition; CE = cannabinoid extract; CRP = C reactive protein; CRT = chemoradiotherapy; DHA = docosahexaenoic acid; EAA = essential amino acids, EE = Ethanwell/Ethanzyme; EPA = eicosapentaenoic acid; FFA = free fatty acid; FFM = fat-free mass; FO = fish oil; GIC = gastrointestinal cancer; IL = interleukin; INF-γ = interferon gamma; LBM = lean body mass; LCTs = long-chain triglycerides; ω-3 LC PUFAs = long chain polyunsaturated fatty acids; MA = megestrol acetate; MCTs = medium-chain triglycerides; MPL = marine phospholipids; ND = normal diet; NI = nutrition intervention; OA = oleic acid; ONS = oral nutrition supplementation; PL = placebo; PMA = psoas major muscle area; QoL = quality of life; REE = resting energy expenditure; SMM = skeletal muscle mass; TAG = triacylglycerols; THC = delta-9-tetrahydrocannabinol; TPN = total parenteral nutrition; βHMB = β-hydroxy-beta-methyl butyrate.

**Table 2:** The dynamics of patients’ body weight after nutritional intervention in the studied clinical trials.

Study ID	Intervention	*n* *	Age (Years)	Baseline BMI (kg/m^2^)	Effect on BW	*p* **
Akita et al., 2019 [36]	EPA	31	67.8 ± 10.7	22.3 ± 2.39	Decrease	0.01
Barber et al., 1999 [37]	EPA	20	62 (51–75)	19.8 (17.8–21.8)	Increase	0.03
Bayram et al., 2009 [38]	EPA	33	7.7 ± 2.7	-	Decrease	NS
Berk et al., 2008 [39]	3 g of βHMB, 14 g arginine, and 14 g of glutamine	235	67 (23–91)	-	Increase	NS
Bruera et al., 2003 [40]	FO	46	63.0 ± 9.1	-	Neutral	NS
Burden et al., 2017 [41]	250 mL/day ONS and dietary advice	55	70.5 ± 11.66	-	Neutral	NS
Candela et al., 2011 [42]	EPA	16	61.31 ± 12.07	20.94 ± 3.72	Neutral	NS
Engelen et al., 2015 [43]	EAA, high leucine mixture	13	68.5 ± 2.1	26.5 ± 1.1	N/A	N/A
Hanai et al., 2018 [44]	ω-3 fatty acids	14	61.5 (45–77)	-	Decrease	N/A
Jatoi et al., 2004 [45]	EPA or MA versus, or both	421 in 3 arms	65 ± 11	-	Neutral	NS
Kraft et al., 2012 [46]	L-carnitine	38	64.4 ± 1.67	24.7 ± 0.65	Increase	0.01
Kun-Yun Yeh et al., 2013 [47]	EE regimen enriched with ω-3 fatty acids, micronutrients, and probiotics	31	54.1± 9.3	20.0 ± 3.1	Increase	<0.05
Martinez et al., 2018 [48]	EPA	32	60± 14	-	Neutral	NS
May et al., 2002 [49]	β-HMB, arginine, and glutamine	18	65.9 ±2.0	-	Increase	<0.05
Palma et al., 2015 [50]	Guarana	18	65 (49–81)	-	Neutral	NS
Persson et al., 2005 [51]	FO and melatonin	13	66 ±9	21.6± 4.1	Increase	NS
Schmidt et al., 2019 [52]	FO capsules	13	68 (59–69)	27.0 (24.1–28.5)	Neutral	NS
	FO nutritional drink group		61 (57–66.8)	25.8 (23.8–27.8)		
Storck et al., 2020 [53]	Leucine-rich supplement (whey protein)	27	62.0 ±11.4	25.0 ±4.6	Increase	NS
Strasser et al., 2006 [54]	CE and THC	164	61 ± 12	-	Decrease	NS
Szefel et al., 2012 [55]	TPN, LCTs, or LCTs plus MCTs as 50/50.	25	66 ± 11	21 ± 5	Decrease	NS
Tayek et al., 1986 [56]	TPN formula (19% BCAA) and a TPN formula (50% BCAA)	10	59.6 ± 4.6	-	Increase	N/A
Taylor et al., 2009 [57]	MPL	17	62.2 ± 8.9	20.2 ± 3.7	Increase	0.006
Turcott et al., 2018 [58]	Nabilone	9	61.1 ± 10.6	20.9 ± 3.5	Decrease	NS
Werner et al., 2017 [59]	FO supplementation, MPL	31	70.3 ± 8.24	21.3 ± 1.73	Neutral	N/A
Wigmore et al., 2000 [60]	High-purity EPA	26	56 (39–75)	23.2 (21.1–27.4)	Increase	<0.05
Zuijdgeest-Van Leeuwen et al., 2000 [61]	EPA-EE or OA-EE	17	64 ± 10	22 ± 3	N/A	N/A

Abbreviations: *n* * = number of patients included in the experimental arm; *p* ** = statistical significance compared to placebo or control group, *p* < 0.05 statistical significance threshold; NS = statistical not significant; N/A = data not available; BCAA = branched-chain amino acid; CE = cannabinoid extract; EAA = essential amino acids; EE = Ethanwell/Ethanzyme; EPA = eicosapentaenoic acid; FO = fish oil; MA = megestrol acetate; MCTs = medium-chain triglycerides; MPL = marine phospholipids; OA = oleic acid; ONS = oral nutrition supplementation; THC = delta-9-tetrahydrocannabinol; TPN = total parenteral nutrition; βHMB = β-hydroxy-beta-methyl butyrate.

## Data Availability

Not applicable.

## Supplementary Materials

The Supplementary Materials can be downloaded at: https://www.mdpi.com/article/10.3390/medicina58070966/s1.

## References

[1] Nishikawa H., Goto M., Fukunishi S., Asai A., Nishiguchi S., Higuchi K. (2021). Cancer Cachexia: Its Mechanism and Clinical Significance. Int. J. Mol. Sci..

[2] Esper D.H., Harb W.A. (2005). The cancer cachexia syndrome: A review of metabolic and clinical manifestations. Nutr Clin. Pract..

[3] Mason M.C., Garcia J.M., Sansgiry S., Walder A., Berger D.H., Anaya D.A. (2016). Preoperative cancer cachexia and short-term outcomes following surgery. J. Surg. Res..

[4] Argilés J.M., Busquets S., Stemmler B., López-Soriano F.J. (2014). Cancer cachexia: Understanding the molecular basis. Nat. Rev. Cancer.

[5] Ni J., Zhang L. (2020). Cancer Cachexia: Definition, Staging, and Emerging Treatments. Cancer Manag. Res..

[6] Bennani-Baiti N., Walsh D. (2009). What is cancer anorexia-cachexia syndrome? A historical perspective. J. R. Coll Physicians Edinb..

[7] Fearon K., Strasser F., Anker S.D., Bosaeus I., Bruera E., Fainsinger R.L., Jatoi A., Loprinzi C., MacDonald N., Mantovani G. (2011). Definition and classification of cancer cachexia: An international consensus. Lancet Oncol..

[8] Cederholm T., Barazzoni R., Austin P., Ballmer P., Biolo G., Bischoff S.C., Compher C., Correia I., Higashiguchi T., Holst M. (2017). ESPEN guidelines on definitions and terminology of clinical nutrition. Clin. Nutr..

[9] Peterson S.J., Mozer M. (2017). Differentiating Sarcopenia and Cachexia among Patients with Cancer. Nutr. Clin. Pract..

[10] Berardi E., Madaro L., Lozanoska-Ochser B., Adamo S., Thorrez L., Bouche M., Coletti D. (2021). A Pound of Flesh: What Cachexia Is and What It Is Not. Diagnostics.

[11] Nicolini A., Ferrari P., Masoni M.C., Fini M., Pagani S., Giampietro O., Carpi A. (2013). Malnutrition, anorexia and cachexia in cancer patients: A mini-review on pathogenesis and treatment. Biomed. Pharm..

[12] Tijerina A.J. (2004). The biochemical basis of metabolism in cancer cachexia. Dimens Crit. Care Nurs..

[13] Montalvo R.N., Hardee J.P., VanderVeen B.N., Carson J.A. (2018). Resistance Exercise’s Ability to Reverse Cancer-Induced Anabolic Resistance. Exerc. Sport Sci. Rev..

[14] Davis M.P., Dreicer R., Walsh D., Lagman R., LeGrand S.B. (2004). Appetite and cancer-associated anorexia: A review. J. Clin. Oncol..

[15] McGovern J., Dolan R.D., Skipworth R.J., Laird B.J., McMillan D.C. (2022). Cancer cachexia: A nutritional or a systemic inflammatory syndrome?. Br. J. Cancer.

[16] Dev R. (2019). Measuring cachexia-diagnostic criteria. Ann. Palliat. Med..

[17] Talbert E.E., Guttridge D.C. (2022). Emerging signaling mediators in the anorexia-cachexia syndrome of cancer. Trends Cancer.

[18] Arends J., Bachmann P., Baracos V., Barthelemy N., Bertz H., Bozzetti F., Fearon K., Hütterer E., Isenring E., Kaasa S. (2017). ESPEN guidelines on nutrition in cancer patients. Clin. Nutr..

[19] Reber E., Schönenberger K.A., Vasiloglou M.F., Stanga Z. (2021). Nutritional Risk Screening in Cancer Patients: The First Step Toward Better Clinical Outcome. Front. Nutr..

[20] Jager-Wittenaar H., Ottery F.D. (2017). Assessing nutritional status in cancer: Role of the Patient-Generated Subjective Global Assessment. Curr. Opin. Clin. Nutr. Metab. Care.

[21] Read J.A., Crockett N., Volker D.H., MacLennan P., Choy S.T.B., Beale P., Clarke S.J. (2005). Nutritional assessment in cancer: Comparing the Mini-Nutritional Assessment (MNA) with the scored Patient-Generated Subjective Global Assessment (PGSGA). Nutr. Cancer.

[22] Boléo-Tomé C., Monteiro-Grillo I., Camilo M., Ravasco P. (2012). Validation of the Malnutrition Universal Screening Tool (MUST) in cancer. Br. J. Nutr..

[23] Sanson G., Sadiraj M., Barbin I., Confezione C., De Matteis D., Boscutti G., Zaccari M., Zanetti M. (2020). Prediction of early- and long-term mortality in adult patients acutely admitted to internal medicine: NRS-2002 and beyond. Clin. Nutr..

[24] Arribas L., Hurtós L., Sendrós M.J., Peiró I., Salleras N., Fort E., Sánchez-Migallón J.M. (2017). NUTRISCORE: A new nutritional screening tool for oncological outpatients. Nutrition.

[25] Vagnildhaug O.M., Blum D., Wilcock A., Fayers P., Strasser F., Baracos V.E., Hjermstad M.J., Kaasa S., Laird B., Solheim T.S. (2017). The applicability of a weight loss grading system in cancer cachexia: A longitudinal analysis. J. Cachexia Sarcopenia Muscle.

[26] McMillan D.C. (2013). The systemic inflammation-based Glasgow Prognostic Score: A decade of experience in patients with cancer. Cancer Treat. Rev..

[27] Purcell S.A., Elliott S.A., Baracos V.E., Chu Q.S., Prado C.M. (2016). Key determinants of energy expenditure in cancer and implications for clinical practice. Eur. J. Clin. Nutr..

[28] Cederholm T., Jensen G.L., Correia M.I.T.D., Gonzalez M.C., Fukushima R., Higashiguchi T., Baptista G., Barazzoni R., Blaauw R., Coats A.J. (2019). GLIM criteria for the diagnosis of malnutrition—A consensus report from the global clinical nutrition community. Clin. Nutr..

[29] Argilés J.M., López-Soriano F.J., Toledo M., Betancourt A., Serpe R., Busquets S. (2011). The cachexia score (CASCO): A new tool for staging cachectic cancer patients. J. Cachexia Sarcopenia Muscle.

[30] Zhou T., Wang B., Liu H., Yang K., Thapa S., Zhang H., Li L., Yu S. (2018). Development and validation of a clinically applicable score to classify cachexia stages in advanced cancer patients. J. Cachexia Sarcopenia Muscle.

[31] Arends J., Baracos V., Bertz H., Bozzetti F., Calder P.C., Deutz N.E.P., Erickson N., Laviano A., Lisanti M.P., Lobo D.N. (2017). ESPEN expert group recommendations for action against cancer-related malnutrition. Clin. Nutr..

[32] Tanaka K., Nakamura S., Narimatsu H. (2022). Nutritional Approach to Cancer Cachexia: A Proposal for Dietitians. Nutrients.

[33] Page M.J., McKenzie J.E., Bossuyt P.M., Boutron I., Hoffmann T.C., Mulrow C.D., Shamseer L., Tetzlaff J.M., Akl E.A., Brennan S.E. (2021). The PRISMA 2020 statement: An updated guideline for reporting systematic reviews. BMJ.

[34] (2020). Review Manager Web (RevMan Web). Version (5.3). The Cochrane Collaboration. revman.cochrane.org.

[35] Higgins J.P.T., Sterne J.A.C. The Cochrane Collaboration. Cochrane Handbook for Systematic Reviews of Interventions Version 5.1.0 2011. www.cochrane-handbook.org.

[36] Akitaae H., Takahashia H., Asukaia K., Tomokunia A., Wadaa H., Marukawab S., Yamasakib T., Yanagimotoa Y., Takahashia Y., Sugimura K. (2019). The utility of nutritional supportive care with an eicosapentaenoic acid (EPA)-enriched nutrition agent during pre-operative chemoradiotherapy for pancreatic cancer: Prospective randomized control study. Clin. Nutr. ESPEN..

[37] Barber M.D., Ross J.A., Voss A.C., Tisdale M.J., Fearon K.C. (1999). The effect of an oral nutritional supplement enriched with fish oil on weight-loss in patients with pancreatic cancer. Br. J. Cancer.

[38] Bayram I., Erbey F., Celik N., Nelson J.L., Tanyeli A. (2009). The use of a protein and energy dense eicosapentaenoic acid containing supplement for malignancy-related weight loss in children. Pediat. Blood Cancer.

[39] Berk L., James J., Schwartz A., Hug E., Mahadevan A., Samuels M., Kachnic L. (2008). A randomized, double-blind, placebo-controlled trial of a beta-hydroxyl beta-methyl butyrate, glutamine, and arginine mixture for the treatment of cancer cachexia (RTOG 0122). Support Care Cancer.

[40] Bruera E., Strasser F., Palmer J.L., Willey J., Calder K., Amyotte G., Baracos V. (2003). Effect of fish oil on appetite and other symptoms in patients with advanced cancer and anorexia/cachexia: A double-blind, placebo-controlled study. J. Clin. Oncol..

[41] Burden S.T., Gibson D.J., Lal S., Hill J., Pilling M., Soop M., Ramesh A., Todd C. (2017). Pre-operative oral nutritional supplementation with dietary advice versus dietary advice alone in weight-losing patients with colorectal cancer: Single-blind randomized controlled trial. J. Cachex Sarcopenia Muscle.

[42] Gomez-Candela C., Sanz M., Horrisberger A., Kohen V., Bermejo L., Auñón P. (2011). Efficacy evaluation of an oral powder supplement enriched with eicosapentaenoic acid in cancer patients. Nutr. Hosp. Organo Of. Soc. Española Nutr. Parenter. Enter..

[43] Engelen M.P.K.J., Safar A.M., Bartter T., Koeman F., Deutz N.E.P. (2015). High anabolic potential of essential amino acid mixtures in advanced nonsmall cell lung cancer. Ann. Oncol..

[44] Hanai N., Terada H., Hirakawa H., Suzuki H., Nishikawa D., Beppu S., Hasegawa Y. (2018). Prospective randomized investigation implementing immunonutritional therapy using a nutritional supplement with a high blend ratio of ω-3 fatty acids during the perioperative period for head and neck carcinomas. Jpn J. Clin. Oncol..

[45] Jatoi A., Rowland K., Loprinzi C.L., Sloan J.A., Dakhil S.R., Macdonald N., Gagnon B., Novotny P.J., Mailliard J.A., Bushey T.I. (2004). An eicosapentaenoic acid supplement versus megestrol acetate versus both for patients with cancer-associated wasting: A North Central Cancer Treatment Group and National Cancer Institute of Canada collaborative effort. J. Clin. Oncol..

[46] Kraft M., Kraft K., Gärtner S., Mayerle J., Simone G., Weber E., Schütte K., Stieler J., Koula-Jenik H., Holzhauer P. (2012). L-Carnitine-supplementation in advanced pancreatic cancer (CARPAN)—A randomized multicentre trial. Nutr. J..

[47] Yeh K.Y., Wang H.M., Chang J.W., Huang J.S., Lai C.H., Lan Y.J., Wu T.H., Chang P.H., Wang H., Wu C.J. (2013). Omega-3 fatty acid-, micronutrient-, and probiotic-enriched nutrition helps body weight stabilization in head and neck cancer cachexia. Oral Surg. Oral Med. Oral Pathol. Oral Radiol..

[48] Solís-Martínez O., Plasa-Carvalho V., Phillips-Sixtos G., Trujillo-Cabrera Y., Hernández-Cuellar A., Queipo-García G.E., Meaney-Mendiolea E., Ceballos G., Fuchs-Tarlovsky V. (2018). Effect of Eicosapentaenoic Acid on Body Composition and Inflammation Markers in Patients with Head and Neck Squamous Cell Cancer from a Public Hospital in Mexico. Nutr. Cancer.

[49] May P.E., Barber A., D’Olimpio J.T., Hourihane A., Abumrad N.N. (2002). Reversal of cancer-related wasting using oral supplementation with a combination of beta-hydroxy-beta-methylbutyrate, arginine, and glutamine. Am. J. Surg..

[50] Palma C.G.L., Lera A.T., Lerner T., De Oliveira M.M., De Borta T.M., Barbosa R.P., Brito G.M., Guazzelli C.A., Cruz F.J.M., del Giglio A. (2015). Guarana (*Paullinia cupana*) Improves Anorexia in Patients with Advanced Cancer. J. Diet. Suppl..

[51] Persson C., Glimelius B., Rönnelid J., Nygren P. (2005). Impact of fish oil and melatonin on cachexia in patients with advanced gastrointestinal cancer: A randomized pilot study. Nutrition.

[52] Schmidt N., Møller G., Bæksgaard L., Østerlind K., Stark K.D., Lauritzen L., Andersen J.R. (2020). Fish oil supplementation in cancer patients. Capsules or nutritional drink supplements? A controlled study of compliance. Clin. Nutr. ESPEN.

[53] Storck L.J., Ruehlin M., Gaeumann S., Gisi D., Schmocker M., Meffert P.J., Imoberdorf R., Pless M., Ballmer P.E. (2020). Effect of a leucine-rich supplement in combination with nutrition and physical exercise in advanced cancer patients: A randomized controlled intervention trial. Clin. Nutr..

[54] Strasser F., Lüftner D., Possinger K., Ernst G., Ruhstaller T., Meissner W., Ko Y.-D., Schnelle M., Reif M., Cerny T. (2006). Comparison of Orally Administered Cannabis Extract and Delta-9-Tetrahydrocannabinol in Treating Patients With Cancer-Related Anorexia-Cachexia Syndrome: A Multicenter, Phase III, Randomized, Double-Blind, Placebo-Controlled Clinical Trial From the Cannabis-InCachexia-Study-Group. J. Clin. Oncol..

[55] Szefel J., Kruszewski W.J., Ciesielski M., Szajewski M., Kawecki K., Aleksandrowicz-Wrona E., Jankun J., Łysiak-Szydłowska W. (2012). L-carnitine and cancer cachexia. I. L-carnitine distribution and metabolic disorders in cancer cachexia. Oncol. Rep..

[56] Tayek J.A., Bistrian B.R., Hehir D.J., Martin R., Moldawer L.L., Blackburn G.L. (1986). Improved protein kinetics and albumin synthesis by branched chain amino acid-enriched total parenteral nutrition in cancer cachexia. A prospective randomized crossover trial. Cancer.

[57] Taylor L.A., Pletschen L., Arends J., Unger C., Massing U. (2010). Marine phospholipids—A promising new dietary approach to tumor-associated weight loss. Support Care Cancer.

[58] Turcott J.G., del Rocío Guillen Núñez M., Flores-Estrada D., Oñate-Ocaña L.F., Zatarain-Barrón Z.L., Barrón F., Arrieta O. (2018). The effect of nabilone on appetite, nutritional status, and quality of life in lung cancer patients: A randomized, double-blind clinical trial. Support Care Cancer.

[59] Werner K., De Gaudry D.K., Taylor L.A., Keck T., Unger C., Hopt U.T., Massing U. (2017). Dietary supplementation with n-3-fatty acids in patients with pancreatic cancer and cachexia: Marine phospholipids versus fish oil—A randomized controlled double-blind trial. Lipids Health Dis..

[60] Wigmore S.J., Barber M.D., Ross J.A., Tisdale M.J., Fearon K.C.H. (2000). Effect of Oral Eicosapentaenoic Acid on Weight Loss in Patients With Pancreatic Cancer. Nutrition and cancer. Nutr. Cancer.

[61] Zuijdgeest-Van Leeuwen S.D., Dagnelie P.C., Wattimena J.L., Van den Berg J.W., van der Gaast A., Swart G.R., Wilson J.F. (2000). Eicosapentaenoic acid ethyl ester supplementation in cachectic cancer patients and healthy subjects: Effects on lipolysis and lipid oxidation. Clin. Nutr..

[62] Barber M.D., Fearon K.C., Tisdale M.J., McMillan D.C., Ross J.A. (2001). Effect of a fish oil-enriched nutritional supplement on metabolic mediators in patients with pancreatic cancer cachexia. Nutr. Cancer.

[63] Hussey H.J., Tisdale M.J. (1999). Effect of a cachectic factor on carbohydrate metabolism and attenuation by eicosapentaenoic acid. Br. J. Cancer.

[64] Lorite M.J., Cariuk P., Tisdale M.J. (1997). Induction of muscle protein degradation by a tumour factor. Br. J. Cancer.

[65] Tisdale M.J., Beck S.A. (1991). Inhibition of tumour-induced lipolysis in vitro and cachexia and tumour growth in vivo by eicosapentaenoic acid. Biochem. Pharm..

[66] Lorite M.J., Thompson M.G., Drake J.L., Carling G., Tisdale M.J. (1998). Mechanism of muscle protein degradation induced by a cancer cachectic factor. Br. J. Cancer.

[67] Whitehouse A.S., Tisdale M.J. (2003). Increased expression of the ubiquitin-proteasome pathway in murine myotubes by proteolysis-inducing factor (PIF) is associated with activation of the transcription factor NF-kappaB. Br. J. Cancer.

[68] Magee P., Pearson S., Allen J. (2008). The omega-3 fatty acid, eicosapentaenoic acid (EPA), prevents the damaging effects of tumour necrosis factor (TNF)-alpha during murine skeletal muscle cell differentiation. Lipids Health Dis..

[69] Nicastro H., Da Luz C.R., Chaves D.F., Bechara L.R., Voltarelli V.A., Rogero M.M., Lancha A.H. (2012). Does Branched-Chain Amino Acids Supplementation Modulate Skeletal Muscle Remodeling through Inflammation Modulation? Possible Mechanisms of Action. J. Nutr. Metab..

[70] Eley H.L., Russell S.T., Tisdale M.J. (2007). Effect of branched-chain amino acids on muscle atrophy in cancer cachexia. Biochem. J..

