# Supplementary material for: Nutritional Interventions to Improve Cachexia Outcomes in Cancer—A Systematic Review
Source: Medicina (Kaunas). 2022 Jul 21;58(7):966. doi: 10.3390/medicina58070966 (PMC9318456; doi:10.3390/medicina58070966)
Supplement: Supplementary file 1 [file medicina-58-00966-s001.zip › medicina-1772488-supplementary.pdf]

Characteristics of studies

Characteristics of included studies

Akita et al. 2019

**Methods**	prospective randomized<br>control study
**Participants**	62 patients randomly<br>assigned to either a nutrition intervention (NI) group (n = 31) or a normal diet (ND) group (n =31)
**Interventions**	eicosapentaenoic acid (EPA)
**Outcomes**	the pre-to-post<br>ratios (post/pre) of skeletal muscle mass and PMA, the post/pre ratios of other nutritional parameters (serum pre-albumin, serum albumin, BMI, and lymphocyte count, and the severity of treatment-related toxicity)
**Notes**	

Risk of bias table

Bias	Authors' judgement	Support for judgement
Random sequence generation (selection bias)	Low risk	stratified by the severity of diabetes mellitus and Glasgow Prognostic Score
Allocation concealment (selection bias)	Low risk	random allocation
Blinding of participants and personnel (performance bias)	High risk	open label
Blinding of outcome assessment (detection bias)	High risk	open label
Incomplete outcome data (attrition bias)	Low risk	data is recorded for all patients
Selective reporting (reporting bias)	Low risk	all prespecified outcomes were reported
Other bias	High risk	31 patients in the treatment arm

Barber et al. 1999

**Methods**	clinical trial
**Participants**	20 patients
**Interventions**	eicosapentaenoic acid (EPA)

**Outcomes**	weight gain (weight, body composition, dietary intake, resting energy expenditure (REE) and<br>performance status)
**Notes**	

Risk of bias table

Bias	Authors' judgement	Support for judgement
Random sequence generation (selection bias)	High risk	no randomization
Allocation concealment (selection bias)	Low risk	patients with unresectable pancreatic adenocarcinoma
Blinding of participants and personnel (performance bias)	High risk	open label
Blinding of outcome assessment (detection bias)	High risk	open label
Incomplete outcome data (attrition bias)	Low risk	data is recorded for all patients
Selective reporting (reporting bias)	Low risk	all prespecified outcomes were reported
Other bias	High risk	20 patients included

Bayram et al. 2009

**Methods**	prospective, randomized, single-center, open label study
**Participants**	52 pediatric patients
**Interventions**	eicosapentaenoic acid (EPA)
**Outcomes**	body weight, body mass index, and weight percentile
**Notes**	

Risk of bias table

Bias	Authors' judgement	Support for judgement
Random sequence generation (selection bias)	Low risk	2:1 randomization scheme
Allocation concealment (selection bias)	Low risk	random allocation
Blinding of participants and personnel (performance bias)	High risk	open label
Blinding of outcome assessment (detection bias)	High risk	open label

Incomplete outcome data (attrition bias)	Low risk	data is recorded for all patients
Selective reporting (reporting bias)	Low risk	all prespecified outcomes were reported
Other bias	High risk	33 patients in the treatment arm

Berk et al. 2008

**Methods**	randomized, double-blind, placebo-controlled trial
**Participants**	472 patients
**Interventions**	3 g of HMB, 14 g arginine, and 14 g of glutamine or<br>placebo - an isonitrogenous, isocaloric mixture to the HMB/Arg/Gln containing 7.72 g l-alanine, 4.28 g glycine, 2.96 g l-serine, 1.23 g l-glutamic acid, and 30.52 g gelatin taken twice a day for 8 weeks
**Outcomes**	the percent of change in lean body mass, body plethysmography, weight, the Schwartz Fatigue Scale, and the Spitzer Quality of Life Scale
**Notes**	

Risk of bias table

**Bias**	**Authors' judgement**	**Support for judgement**
Random sequence generation (selection bias)	Unclear risk	not specified
Allocation concealment (selection bias)	Low risk	random allocation
Blinding of participants and personnel (performance bias)	Low risk	double blind
Blinding of outcome assessment (detection bias)	Low risk	double blind
Incomplete outcome data (attrition bias)	Low risk	data is recorded for all patients
Selective reporting (reporting bias)	Low risk	all prespecified outcomes were reported
Other bias	Unclear risk	235 patients in the treatment arm

Bruera et al. 2003

**Methods**	randomized, double-blind, placebo-controlled trial
**Participants**	91 patients
**Interventions**	180 mg of eicosapentaenoic acid (EPA), 120 mg<br>of docosahexaenoic acid [DHA], and 1 mg of vitamin E or placebo - olive oil

**Outcomes**	Appetite, tiredness, nausea, well-being, caloric intake, nutritional status, and function
**Notes**	

Risk of bias table

Bias	Authors' judgement	Support for judgement
Random sequence generation (selection bias)	Unclear risk	not specified
Allocation concealment (selection bias)	Low risk	random allocation
Blinding of participants and personnel (performance bias)	Low risk	double blind
Blinding of outcome assessment (detection bias)	Low risk	double blind
Incomplete outcome data (attrition bias)	Low risk	data is recorded for all patients
Selective reporting (reporting bias)	Low risk	all prespecified outcomes were reported
Other bias	High risk	46 patients in the treatment arm

Burden et al. 2017

**Methods**	single-blind randomized controlled trial
**Participants**	101 patients
**Interventions**	Intervention group was given 250 mL/day oral nutrition supplements (10.1 KJ and 0.096 g protein per mL) and dietary advice. Control group received dietary advice alone
**Outcomes**	patients with one or more surgical site infection (SSI) or chest infection; secondary outcomes included percentage weight loss, total complications, and body composition measurements
**Notes**	

Risk of bias table

Bias	Authors' judgement	Support for judgement
Random sequence generation (selection bias)	Low risk	1:1 ratio by using blocks of two ensuring equal numbers in each group
Allocation concealment (selection bias)	Low risk	sequentially numbered opaque sealed envelopes

Blinding of participants and personnel (performance bias)	Low risk	The research team was blind to the intervention, but the participants were not
Blinding of outcome assessment (detection bias)	Low risk	double blind
Incomplete outcome data (attrition bias)	Low risk	data is recorded for all patients
Selective reporting (reporting bias)	Low risk	all prespecified outcomes were reported
Other bias	Unclear risk	55 patients in the treatment arm

Candela et al. 2011

**Methods**	randomized placebo-controlled trial
**Participants**	61 patients
**Interventions**	an oral powder supplement enriched with eicosapentaenoic acid (EPA) compared to a standard liquid supplement
**Outcomes**	the Patient-Generated Subjective Global Assessment (pg-SGA), anthropometric measurements (skin folds, circumferences and bioimpedance), dietary parameters (3-day food record), biochemical and inflammatory parameters (basic biochemistry, cytokines, prealbumin and Reactive C Protein)
**Notes**	

Risk of bias table

**Bias**	**Authors' judgement**	**Support for judgement**
Random sequence generation (selection bias)	Unclear risk	not specified
Allocation concealment (selection bias)	Low risk	random allocation
Blinding of participants and personnel (performance bias)	High risk	open label
Blinding of outcome assessment (detection bias)	High risk	open label
Incomplete outcome data (attrition bias)	Low risk	data is recorded for all patients
Selective reporting (reporting bias)	Low risk	all prespecified outcomes were reported
Other bias	High risk	16 patients in the treatment arm

Engelen et al. 2015

**Methods**	randomized double blind cross-over design
**Participants**	24 patients
**Interventions**	14 g of free EAA with high leucine levels (EAA/leucine) versus a balanced amino acid mixture containing both EAA and non-EAA as present in whey protein
**Outcomes**	Body weight, height, fat, and fat-free mass (FFM), respiratory muscle function, handgrip strength, and endurance. Protein anabolism
**Notes**	

Risk of bias table

**Bias**	**Authors' judgement**	**Support for judgement**
Random sequence generation (selection bias)	Unclear risk	not specified
Allocation concealment (selection bias)	Low risk	random allocation
Blinding of participants and personnel (performance bias)	Low risk	double blind cross-over design
Blinding of outcome assessment (detection bias)	Low risk	double blind cross-over design
Incomplete outcome data (attrition bias)	Low risk	data is recorded for all patients
Selective reporting (reporting bias)	Low risk	all prespecified outcomes were reported
Other bias	High risk	13 patients in the treatment arm

Hanai et al. 2018

**Methods**	prospective randomized controlled study
**Participants**	28 patients
**Interventions**	EPA-enriched oral nutritional supplement (Prosure®) or no dietary intervention
**Outcomes**	the postoperative nutritional status (weight, lean body mass, albumin, prealbumin),<br><br>while the secondary endpoints included inflammatory<br>marker levels (CRP, IL6, white blood cell count, body temperature), compliance with the Prosure® dosage, and the occurrence of postoperative complications.
**Notes**	

Risk of bias table

Bias	Authors' judgement	Support for judgement
Random sequence generation (selection bias)	Unclear risk	not specified
Allocation concealment (selection bias)	Low risk	random allocation
Blinding of participants and personnel (performance bias)	High risk	open label
Blinding of outcome assessment (detection bias)	High risk	open label
Incomplete outcome data (attrition bias)	Low risk	data is recorded for all patients
Selective reporting (reporting bias)	Low risk	all prespecified outcomes were reported
Other bias	High risk	14 patients in the treatment arm

Jatoi et al. 2004

**Methods**	double-blinded, placebo-controlled trial
**Participants**	421 patients
**Interventions**	an EPA supplement 1.09 g administered bid plus placebo; MA liquid suspension 600 mg/d plus an isocaloric, isonitrogenous supplement administered twice a day; or both
**Outcomes**	a 10% weight gain above baseline
**Notes**	

Risk of bias table

Bias	Authors' judgement	Support for judgement
Random sequence generation (selection bias)	Low risk	The stratification process used here was a minimization algorithm that balances the marginal distributions
Allocation concealment (selection bias)	Low risk	random allocation
Blinding of participants and personnel (performance bias)	Low risk	double blind
Blinding of outcome assessment (detection bias)	Low risk	double blind

Incomplete outcome data (attrition bias)	Low risk	data is recorded for all patients
Selective reporting (reporting bias)	Low risk	all prespecified outcomes were reported
Other bias	Low risk	more than 200 patients in the treatment arm

Kraft et al. 2012

**Methods**	prospective, multi-centre, placebo-controlled, randomized and double-blinded trial
**Participants**	72 patients
**Interventions**	oral L-Carnitine (4 g) or placebo for 12 weeks
**Outcomes**	adverse effects, quality of life, fatigue, BMI, body composition, survival time, changes in L-carnitine level, CRP, albumine, leucocytes, CA 19-9
**Notes**	

Risk of bias table

**Bias**	**Authors' judgement**	**Support for judgement**
Random sequence generation (selection bias)	Low risk	sequential series of 4 per block
Allocation concealment (selection bias)	Low risk	computer<br>generated randomization code, sealed envelopes
Blinding of participants and personnel (performance bias)	Low risk	double blind
Blinding of outcome assessment (detection bias)	Low risk	double blind
Incomplete outcome data (attrition bias)	Low risk	data is recorded for all patients
Selective reporting (reporting bias)	Low risk	all prespecified outcomes were reported
Other bias	High risk	38 patients in the treatment arm

Kun-Yun Yeh et al 2013

**Methods**	prospective randomized placebo-controlled clinical trial
**Participants**	68 patients
**Interventions**	an Ethanwell/Ethanzyme (EE) regimen enriched with omega-3 fatty acids, micronutrients, and probiotics, or control (Isocal) for a 3-month period
**Outcomes**	body weight (BW) changes, serum albumin and prealbumin levels
**Notes**	

Risk of bias table

Bias	Authors' judgement	Support for judgement
Random sequence generation (selection bias)	Low risk	1:1 ratio
Allocation concealment (selection bias)	Low risk	random allocation, sealed envelopes
Blinding of participants and personnel (performance bias)	Unclear risk	not specified
Blinding of outcome assessment (detection bias)	Unclear risk	not specified
Incomplete outcome data (attrition bias)	Low risk	data is recorded for all patients
Selective reporting (reporting bias)	Low risk	all prespecified outcomes were reported
Other bias	High risk	31 patients in the treatment arm

Martinez et al. 2018

**Methods**	randomized single-blind placebo-controlled clinical trial
**Participants**	64 patients
**Interventions**	Eicosapentaenoic acid (EPA)
**Outcomes**	body composition by bioelectrical impedance analysis and determined IL-1b, IL-6, TNF-a and IFN-g, CRP,<br>serum proteins, and blood count at baseline and at the end of the study
**Notes**	

Risk of bias table

Bias	Authors' judgement	Support for judgement
Random sequence generation (selection bias)	Unclear risk	not specified
Allocation concealment (selection bias)	Low risk	random allocation
Blinding of participants and personnel (performance bias)	Low risk	single blind
Blinding of outcome assessment (detection bias)	Unclear risk	single blind
Incomplete outcome data (attrition bias)	Low risk	data is recorded for all patients
Selective reporting (reporting bias)	Low risk	all prespecified outcomes were reported
Other bias	High risk	32 patients in the treatment arm

May et al. 2002

**Methods**	randomized double-blind placebo-controlled clinical trial
**Participants**	49 patients
**Interventions**	oral supplementation with a<br>combination of hydroxy--methylbutyrate, arginine, and glutamine or an isonitrogenous control mixture of nonessential amino acids
**Outcomes**	the change in body mass and fat-free mass (FFM)
**Notes**	

Risk of bias table

Bias	Authors' judgement	Support for judgement
Random sequence generation (selection bias)	Low risk	computer-generated<br>random numbers
Allocation concealment (selection bias)	Low risk	computer-generated<br>random numbers prior to the start of the study in a doubleblind<br>fashion
Blinding of participants and personnel (performance bias)	Low risk	double blind
Blinding of outcome assessment (detection bias)	Unclear risk	double blind
Incomplete outcome data (attrition bias)	Low risk	data is recorded for all patients
Selective reporting (reporting bias)	Low risk	all prespecified outcomes were reported
Other bias	High risk	18 patients in the treatment arm

Palma et al. 2015

**Methods**	clinical trial (Simon model)
**Participants**	30 patients
**Interventions**	50 mg of the crude dry extract<br>of guarana twice a day for 4 weeks
**Outcomes**	a positive response in the first phase to be at least 5% weight gain or a three-point improvement in the appetite scale in at least three of the first 18 evaluable patients
**Notes**	

Risk of bias table

Bias	Authors' judgement	Support for judgement
Random sequence generation (selection bias)	Unclear risk	nonrandomized trial
Allocation concealment (selection bias)	Unclear risk	nonrandomized trial
Blinding of participants and personnel (performance bias)	Unclear risk	open label
Blinding of outcome assessment (detection bias)	Unclear risk	open label
Incomplete outcome data (attrition bias)	Low risk	data is recorded for all patients
Selective reporting (reporting bias)	Low risk	all prespecified outcomes were reported
Other bias	High risk	18 patients completed the protocol

Persson et al. 2005

**Methods**	a one-center, randomized, non-placebo controlled,<br>open study
**Participants**	24 patients
**Interventions**	4.9 g of eicosapentaenoic acid and 3.2 g of docosahexanoic acid, or 18 mg/d of melatonin for 4 week
**Outcomes**	changes in tumor necrosis factor-alpha, interleukin-1beta, soluble interleukin-2 receptor, interleukin-6, and interleukin-8 and the fatty acids: eicosapentaenoic acid, docosahexanoic acid, arachidonic acid, and linoleic acid.
**Notes**	

Risk of bias table

Bias	Authors' judgement	Support for judgement
Random sequence generation (selection bias)	Unclear risk	not specified
Allocation concealment (selection bias)	Low risk	random allocation, non-placebo
Blinding of participants and personnel (performance bias)	High risk	open label
Blinding of outcome assessment (detection bias)	High risk	open label
Incomplete outcome data (attrition bias)	Low risk	data is recorded for all patients

Selective reporting (reporting bias)	Low risk	all prespecified outcomes were reported
Other bias	High risk	13 patients in the fish-oil arm

Schmidt et al. 2019

**Methods**	non-randomized clinical trial
**Participants**	41 patients
**Interventions**	a nutritional drink with fish-oil compared to an equivalent dose of fish-oil administered as<br>capsules
**Outcomes**	changes in whole blood n-3 LC<br>PUFAs, weight, nutritional status, acceptability or side effects
**Notes**	

Risk of bias table

**Bias**	**Authors' judgement**	**Support for judgement**
Random sequence generation (selection bias)	Unclear risk	non-randomized
Allocation concealment (selection bias)	Unclear risk	non-randomized
Blinding of participants and personnel (performance bias)	Unclear risk	open label
Blinding of outcome assessment (detection bias)	Unclear risk	open label
Incomplete outcome data (attrition bias)	Low risk	data is recorded for all patients
Selective reporting (reporting bias)	Low risk	all prespecified outcomes were reported
Other bias	High risk	13 patients included in the analysis

Storck et al. 2020

**Methods**	randomized controlled intervention trial
**Participants**	52 patients
**Interventions**	a leucine-rich supplement in combination with a<br>nutrition and physical exercise program versus standard care
**Outcomes**	changes in physical function, physical performance tests, nutritional status, dietary intake, fatigue, quality of<br>life (QoL) and clinical course

**Notes**	

Risk of bias table

Bias	Authors' judgement	Support for judgement
Random sequence generation (selection bias)	Low risk	a 1:1 ratio using block sizes of six by the data management program<br>secuTrial®
Allocation concealment (selection bias)	Low risk	stratified according to the site of their<br>primary tumor (lung cancer, upper or lower gastrointestinal tract<br>cancer, other)
Blinding of participants and personnel (performance bias)	Unclear risk	not specified
Blinding of outcome assessment (detection bias)	Unclear risk	not specified
Incomplete outcome data (attrition bias)	Low risk	data is recorded for all patients
Selective reporting (reporting bias)	Low risk	all prespecified outcomes were reported
Other bias	High risk	27 patients in the treatment arm

Strasser et al. 2006

**Methods**	a multicenter, phase III, randomized, double-Blind,<br>placebo-controlled clinical trial
**Participants**	243 patients
**Interventions**	cannabis extract (CE), delta-9-tetrahydrocannabinol (THC), and placebo
**Outcomes**	appetite, mood, nausea and quality of life (QOL)
**Notes**	

Risk of bias table

Bias	Authors' judgement	Support for judgement
Random sequence generation (selection bias)	Low risk	Random assignment lists, stratified by center, were prepared by a naive<br>statistician using SAS software
Allocation concealment (selection bias)	Low risk	study drug sets in multiples of five, together with matching sealed envelopes containing individual treatment assignments

Blinding of participants and personnel (performance bias)	Low risk	Investigators remained blinded until the study ended, with individual unblinding permitted only for safety reasons.
Blinding of outcome assessment (detection bias)	Low risk	The statistician and data manager who were managing random assignment, unblinding, and related decisions were naive to clinical evaluations and uninvolved in data management or analysis
Incomplete outcome data (attrition bias)	Low risk	data is recorded for all patients
Selective reporting (reporting bias)	Low risk	all prespecified outcomes were reported
Other bias	Unclear risk	164 patients in the treatment arm

Szefel et al. 2012

**Methods**	randomized clinical trial
**Participants**	50 patients
**Interventions**	total parenteral nutrition (TPN) with long-chain triglycerides (LCTs), or LCTs plus medium-chain triglycerides (MCTs)
**Outcomes**	L-Carnitine distribution and the effects of parenteral lipid emulsions on plasma L-Carnitine levels and urinary excretion
**Notes**	

Risk of bias table

Bias	Authors' judgement	Support for judgement
Random sequence generation (selection bias)	Low risk	50:50
Allocation concealment (selection bias)	Low risk	randomly divided into 2 groups according to the type of lipid emulsion they were administered: MCT/LCT vs. pure LCT.
Blinding of participants and personnel (performance bias)	Unclear risk	not specified
Blinding of outcome assessment (detection bias)	Unclear risk	not specified
Incomplete outcome data (attrition bias)	Low risk	data is recorded for all patients

Selective reporting (reporting bias)	Low risk	all prespecified outcomes were reported
Other bias	High risk	25 patients in the treatment arm

Tayek et al.1986

**Methods**	A prospective randomized crossover trial
**Participants**	10 patients
**Interventions**	a conventional total parenteral nutrition (TPN) formula containing 19% branched chain amino acid (BCAA) and a BCAA-enriched TPN formula containing 50% of the amino acids as BCAA
**Outcomes**	changes in the whole body leucine kinetics and fractional rates of albumin synthesis
**Notes**	

Risk of bias table

**Bias**	**Authors' judgement**	**Support for judgement**
Random sequence generation (selection bias)	Unclear risk	not specified
Allocation concealment (selection bias)	Low risk	random allocation
Blinding of participants and personnel (performance bias)	Unclear risk	not specified
Blinding of outcome assessment (detection bias)	Unclear risk	not specified
Incomplete outcome data (attrition bias)	Low risk	data is recorded for all patients
Selective reporting (reporting bias)	Low risk	all prespecified outcomes were reported
Other bias	High risk	10 patients included in the study

Taylor et al. 2009

**Methods**	clinical trial
**Participants**	31 patients
**Interventions**	marine phospholipids (1.5 g/day) as softgel capsules for a period of 6 weeks
**Outcomes**	compliance, changes in body weight, appetite, and quality of life, fatty acid profile in plasma and blood cells
**Notes**	

Risk of bias table

Bias	Authors' judgement	Support for judgement
Random sequence generation (selection bias)	High risk	non-randomized
Allocation concealment (selection bias)	Unclear risk	no selection
Blinding of participants and personnel (performance bias)	High risk	open label
Blinding of outcome assessment (detection bias)	High risk	open label
Incomplete outcome data (attrition bias)	Low risk	data is recorded for all patients
Selective reporting (reporting bias)	Low risk	all prespecified outcomes were reported
Other bias	High risk	17 patients analyzed

Turcott et al. 2018

**Methods**	a randomized, double-blind, placebo-controlled clinical trial
**Participants**	47 patients
**Interventions**	Nabilone vs. placebo
**Outcomes**	appetite, nutritional status, and quality of life
**Notes**	

Risk of bias table

Bias	Authors' judgement	Support for judgement
Random sequence generation (selection bias)	Unclear risk	not specified
Allocation concealment (selection bias)	Low risk	random allocation
Blinding of participants and personnel (performance bias)	Low risk	double blind
Blinding of outcome assessment (detection bias)	Low risk	double blind
Incomplete outcome data (attrition bias)	Low risk	data is recorded for all patients
Selective reporting (reporting bias)	Low risk	all prespecified outcomes were reported
Other bias	High risk	9 patients in the treatmend arm finished the study

Werner et al. 2017

**Methods**	a randomized controlled double blind trial
**Participants**	60 patients
**Interventions**	marine phospholipids versus<br>fish oil
**Outcomes**	compliance, quality of life, nutritional habits and changes in routine blood parameters,<br>lipid profiles, body weight, and appetite
**Notes**	

Risk of bias table

Bias	Authors' judgement	Support for judgement
Random sequence generation (selection bias)	Low risk	not specified
Allocation concealment (selection bias)	Low risk	Randomization and masking was performed with assignment envelopes containing the letter A<br>or B, which were prepared by a non-involved external party (Membramed GmbH).
Blinding of participants and personnel (performance bias)	Low risk	double blind
Blinding of outcome assessment (detection bias)	Low risk	Assignment code was revealed<br>at the end of the study by Membramed GmbH
Incomplete outcome data (attrition bias)	Low risk	data is recorded for all patients
Selective reporting (reporting bias)	Low risk	all prespecified outcomes were reported
Other bias	High risk	31 patients in the treatment arm

Wigmore et al. 2000

**Methods**	clinical trial
**Participants**	26 patients
**Interventions**	EPA at 1 g/day for the<br>first week, 2 g/day for the second week, 4 g/day for the third week, and 6 g/day thereafter.
**Outcomes**	overall survival, changes in weight, body composition, hematologic and clinical chemistry variables, acute- phase protein response, and performance status

**Notes**	

Risk of bias table

Bias	Authors' judgement	Support for judgement
Random sequence generation (selection bias)	High risk	non-randomization
Allocation concealment (selection bias)	Unclear risk	non-randomization
Blinding of participants and personnel (performance bias)	High risk	open label
Blinding of outcome assessment (detection bias)	High risk	open label
Incomplete outcome data (attrition bias)	Low risk	data is recorded for all patients
Selective reporting (reporting bias)	Low risk	all prespecified outcomes were reported
Other bias	High risk	26 patients in the study

Zuijdgeest-Van Leeuwen et al. 2000

**Methods**	double-blind, randomized trial
**Participants**	33 patients
**Interventions**	EPA-EE (6 g/d) or placebo (oleic acid (OA)-EE; 6 g/d) for seven days
**Outcomes**	whole-body lipolysis and palmitic acid release were measured in the overnight fasting state, changes in weight, plasma free fatty acids, triacylglycerols, CRP, albumin and prealbumin
**Notes**	

Risk of bias table

Bias	Authors' judgement	Support for judgement
Random sequence generation (selection bias)	Unclear risk	not specified
Allocation concealment (selection bias)	Low risk	random allocation
Blinding of participants and personnel (performance bias)	Low risk	double blind
Blinding of outcome assessment (detection bias)	Low risk	double blind
Incomplete outcome data (attrition bias)	Low risk	data is recorded for all patients

Selective reporting (reporting bias)	Low risk	all prespecified outcomes were reported
Other bias	High risk	17 patients in the treatment arm

Footnotes

Characteristics of excluded studies

Footnotes

Characteristics of studies awaiting classification

Footnotes

Characteristics of ongoing studies

Footnotes
